# Enhanced cricket match prediction using kernel methods for feature extraction and back-propagation neural networks

**DOI:** 10.1038/s41598-026-36555-6

**Published:** 2026-01-28

**Authors:** K. Dhinakaran, S. Anbuchelian

**Affiliations:** 1 Department of Computer Science and Engineering, Chennai Institute of Technology (An Autonomous Institution), Sarathy Nagar, Kundrathur, Kanchipuram District, Tamilnadu 600069 India; 2 Ramanujan Computing Centre, College of Engineering Guindy, Anna University, Chennai, 600025 India

**Keywords:** Supervised learning, Cricket prediction, One day international, Classification, Neural networks, Engineering, Mathematics and computing

## Abstract

This study presents a dynamic machine learning framework for predicting the outcome of One Day International (ODI) cricket matches by analysing match progression at multiple game states. Each over is treated as a distinct match state, enabling real-time outcome prediction throughout the innings. Six key criteria are employed for classification, namely balls remaining, lead of Team A, wickets remaining, relative team strength, home advantage, and toss outcome. Feature extraction is performed using the League Championship Algorithm (LCA), which selects the most informative features from historical cricket data, followed by classification using a Back-Propagation Neural Network (BPNN). Experimental results demonstrate that the proposed model achieves an accuracy of 83%, a true positive rate of 0.81, a positive predictive value of 0.79, and an F1-score of 0.80 on the validation dataset, outperforming conventional prediction approaches by 5–10% across key performance metrics. The findings confirm the effectiveness of combining optimized feature extraction with neural network-based classification for accurate and interpretable cricket match outcome prediction.

## Introduction

Cricket offers a rich domain for data-driven research due to its strategic depth, complex rules, and continuously evolving match dynamics^1^. Among the various formats of the game, One Day Internationals (ODIs) and Twenty20 (T20) cricket present particularly challenging prediction scenarios because of their limited overs, fluctuating run rates, and frequent momentum shifts. Each team consists of eleven players, and the outcome of a match is influenced by a combination of individual performance, team composition, and contextual factors^2^.

The increasing availability of ball-by-ball cricket datasets has enabled the application of machine learning techniques for accurate match outcome prediction^3^. However, cricket prediction remains challenging due to the interdependence of batting, bowling, and fielding performances, along with external influences such as home advantage, pitch conditions, and toss decisions^4–6^. These factors evolve dynamically during a match, requiring prediction models that can adapt to changing game states in real time^7^.

The problem this paper tackles is the prediction of match results depending on real-time game statistics. Using a Back-Propagation Neural Network (BPNN) model specifically helps one to predict the results of ODI and T20 games. Overs completed and runs scored define the prediction, which is on the likelihood of a team winning at several states over the game. The challenge is to develop a model that fairly forecasts match results based on several criteria, including player statistics and match conditions^8^.

The primary objective of this research is to develop a robust machine learning model for predicting cricket match outcomes using dynamic in-game information. Specifically, this study aims to design a BPNN-based classification framework that integrates comprehensive player statistics, match progression variables, and contextual factors. Another objective is to identify and evaluate the most influential features affecting prediction accuracy through optimized feature extraction^9^. Finally, the proposed approach is compared with existing prediction models, including SHAP-based models, LASSO-SVM, LASSO-XGBoost, and Bayesian Additive Regression Trees (BART), to demonstrate its effectiveness.

The key contributions of this study include the integration of domain-driven feature engineering with LCA-based feature extraction, the application of BPNN for state-wise match outcome prediction, and a comprehensive experimental evaluation across ODI, Test, and T20 formats^10^.

##  Related works

In cricket, new advances in sports analytics show how progressively machine learning techniques are being applied to raise predictive accuracy and strategic decision-making.

The complicated character of cricket shaped by several elements demands sophisticated analytical techniques. Conventional methods such the Current Run Rate (CRR) have limitations, particularly in One Day Internationals (ODIs) where the pace of the game may vary greatly. Researching this area has focused on improving player classifications and score forecasts by means of machine learning. Since these models have shown promise in providing more accurate predictions than traditional approaches, they underline the success of machine learning in performance analysis and decision-making^11^.

Dynamic predictions for T20 games, where game scenarios may vary rapidly, find use for machine learning. A study^12^ dynamically changed predictions as the T20 international match progressed using supervised machine learning approaches, so forecasting the winner. By means of explainable machine learning techniques including SHAP (SHapley Additive exPlanations) scores, the study was able to identify salient features influencing predictions at several phases of the match. This approach displayed more accuracy from about 55% in the first stages to almost 85% in the last phases of the game. These strategies are quite helpful for analysts assessing and adjusting strategies in real-time as well as for coaches.

Building on dynamic forecasts, another study^13^ focused on matching results at several T20 game stages, more especially, within the Indian Premier League (IPL). With an 812 completed match dataset, the LASSO approach for feature selection revealed the most significant characteristics. Classification models were assessed using Naive Bayes, Logistic Regression, and SVM; Naive Bayes showed prediction accuracy over stages ranging from 53.08% to 91.76%. Likewise, SVM and logistic regression (LR) produced like results. This research exposed not only in-game forecasts but also a strategy generator to enable teams to build winning plans based on real-time predictions.

Developing effective plans in T20 cricket depends on accurate first innings score prediction. Research^14^ investigated this using among other regression methods feature engineering and XGBoost, Lasso, and Ridge regression. The study revealed the possibility of these machine learning techniques in forecasting first innings scores by evaluating model performance using measures of mean absolute error, root mean squared error, and R-squared values. Teams aiming high and planning in line with these projections will find them rather useful.

Maximising team performance calls for an awareness of contextual elements including home advantage, throw decisions, and opponent strengths. A^15^ study examined BART emerged as the most effective model after results were examined using partial dependence graphs and accumulated local effects. Applying machine learning in cricket has significantly raised the ability to predict match results, player performance, and strategic decisions. These studies reveal the several ways that they influence strategic planning and accuracy improvement in cricket analytics^16^. Summary are in Table 1.

**Table 1:** Summary.

Method	Algorithm	Methodology	Outcomes
^11^	Linear Regression, Logistic Regression, Naive Bayes, SVM, Decision Tree, Random Forest	Predictive modelling	Enhanced accuracy in predicting cricket scores and player roles
^12^	Supervised Machine Learning, SHAP Scores	Dynamic prediction of match outcomes with real-time feature importance analysis	Accuracy increased from 55% to 85% throughout the match, valuable for real-time decision-making
^13^	Naive Bayes, Logistic Regression, SVM	In-game predictions at various stages using LASSO for feature selection	Prediction accuracy ranged from 53.08% to 97.65% depending on the match stage; introduced strategy generator
^14^	XGBoost, Lasso, Ridge Regression	Historical data analysis with feature engineering for first innings score prediction	Improved accuracy in first innings score prediction, aiding in target-setting strategies
^15^	Gradient Boosting, Regression Tree, Bagging, Random Forest, BART	Analysis of contextual factors using tree-based models and interpretable ML methods	Identified key contextual factors affecting performance; provided strategies for pre-match and pre-season decisions

Current research focusses on individual elements of match prediction and player performance even though they lack full models combining real-time dynamic changes, contextual factors, and holistic team strategies. Research combining these elements would help to develop more robust and flexible prediction systems, so enhancing general decision-making and strategic development in cricket.

## Proposed method

**Fig. 1:**
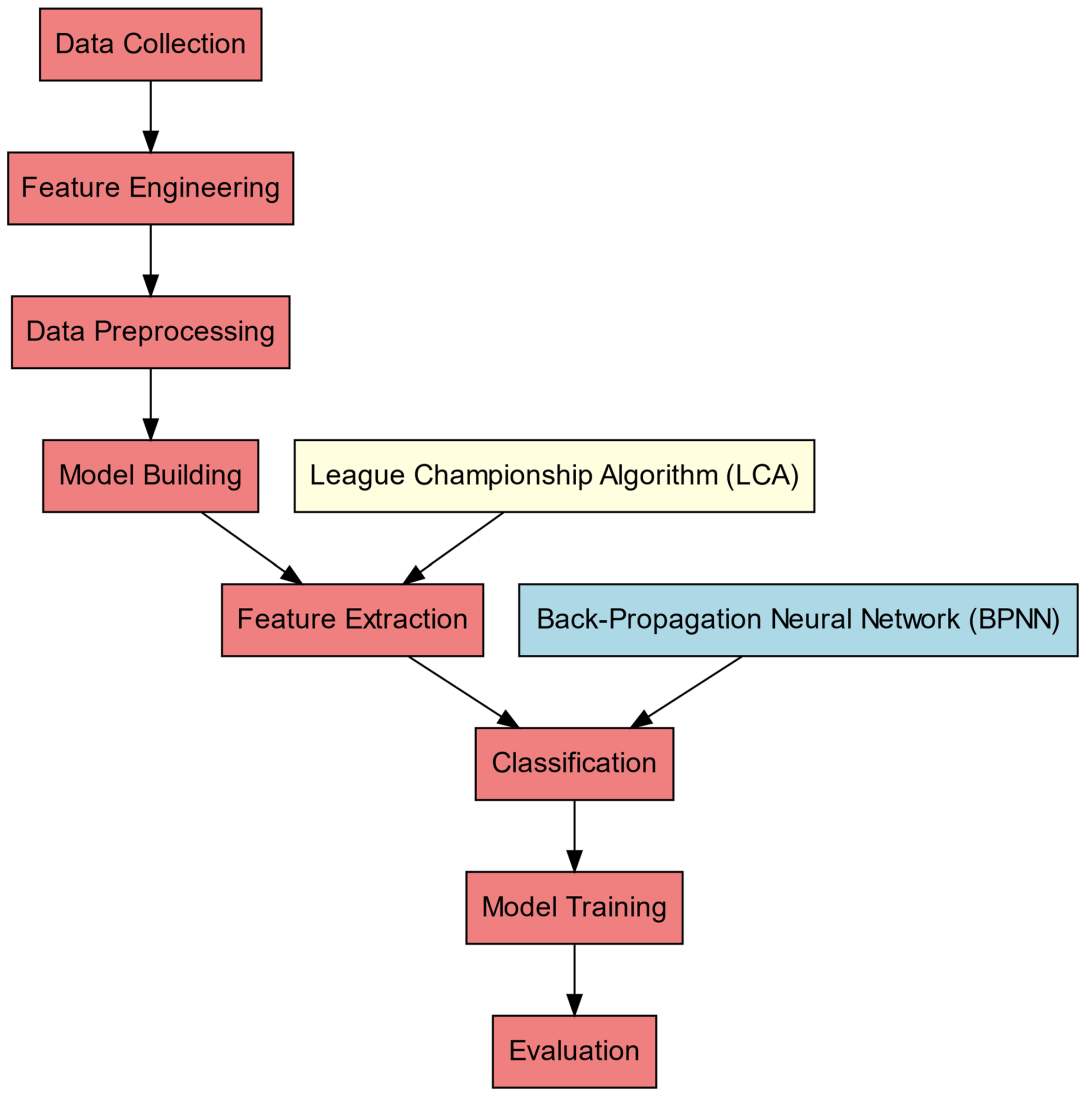
Proposed prediction framework.

The proposed method uses historical cricket data to quantify the relative strength of competing teams, using past performances. Combining raw data with contextual game statistics, the dataset is organised from unstructured form into a structured one including significant elements including batting averages, bowling economy, and fielding statistics. Including balls remaining, lead of Team A, wickets remaining, relative team strength, home advantage, and toss outcome, the model input characteristics combine stationary and dynamic aspects. Every state of the innings is a different data point with the match outcome as the target label; hence, the prediction is handled as a supervised classification problem. Past match data shows the model is trained for feature extraction using the League Championship Algorithm (LCA) and Back-Propagation Neural Network (BPNN). Starting with scoring based on past performance for every player, the feature extraction process aggregates these values to determine the relative team strength. This dynamic, real-time assessment of the match conditions helps to change projections as the game progresses. Proposed Prediction Framework is shown in Fig. 1.

Unlike conventional machine learning pipelines, domain-specific feature construction is performed prior to preprocessing to capture cricket-specific match state variables directly from raw ball-by-ball data. These features are subsequently normalized and refined before LCA-based feature extraction. The optimized feature subset is then used to train the Back-Propagation Neural Network for match outcome classification.

### Pseudocode

# Pseudocode for Cricket Match Outcome Prediction

# Step 1: Data Collection

data = collect_cricket_data(start_year = 1877, end_year = current_year)

# Step 2: Feature Engineering

features = extract_features(data)

# Step 3: Data Preprocessing

preprocessed_data = preprocess_data(features)

# Step 4: Model Building - Feature Extraction using LCA

LCA_features = apply_LCA(preprocessed_data)

# Step 5: Model Training

model = train_BPNN(LCA_features, target_labels)

# Step 6: Evaluation

evaluate_model(model, validation_data)

### Problem statement

To model the problem of predicting the match outcome, we define each innings of a match MMM as a series of states. The first and second innings are represented as I1 and I2​, respectively. Each innings is divided into 50 distinct states Sk​, where 0 ≤ k ≤ 49. Here, Sk​ denotes the state of the innings at the end of the kth over. The goal is to predict the outcome (win or loss) for Team B at each state Sk​ in both innings, dynamically adjusting as the match progresses.

In this study, six fundamental criteria are used to describe the match state: balls remaining, lead of Team A, wickets remaining, relative team strength, home advantage, and toss outcome. Among these, balls remaining, relative team strength, and toss outcome are explicitly formulated in this subsection to illustrate the mathematical modeling process, while the remaining criteria are incorporated as contextual or categorical inputs during classifier training (Fig. 1).

### Mathematical representation

The prediction task can be formulated as a supervised machine learning classification problem. Let the prediction at state S0​ in I1​ represent the situation before the first ball of the match is bowled. At this point, the prediction is made with no deliveries having been bowled, and the state S0​ in I2​ (equivalent to S50 in I1​) represents the end of I1​ or the state before the start of the 1st over in I2​. The outcome prediction focuses on determining whether Team B wins or loses in the state Sk, where 0 ≤ k ≤ 49, for both I1​ and I2​ in a given match MMM. The prediction for each state depends on a set of features that capture both static and dynamic aspects of the match. These features are:

1. Balls remaining (Bk): the number of deliveries remaining at state Sk​ is calculated as
  Bk = 300-(k×6), where  0 ≤ k ≤ 49.
  This formula represents the total number of deliveries minus those bowled by the end of over *k*.
2. Relative team strength (TS): This feature quantifies the strengths of both teams based on the historical performance data of individual players. The strength of a team is computed by aggregating the performance scores of its players, derived from metrics such as batting averages and bowling economy rates.
3. Toss (T): This feature is binary and is set to:

$$T=\left\{ {\begin{array}{*{20}{l}} 1&{{\text{if Team B wins the toss}}} \\ 0&{{\text{if Team A wins the toss}}} \end{array}} \right.$$

### Classification problem

Each data point corresponds to a state Sk​ in innings I1​ or I2​, characterized by the six features discussed above. The target label *Y* for each data point is defined as:

$$Y=\left\{ {\begin{array}{*{20}{l}} 1&{{\text{if Team B wins the match}}} \\ 0&{{\mathrm{otherwise}}} \end{array}} \right.$$

Thus, the task is to train a classifier to predict *Y* based on the feature set (Bk, Lk, Wk, TS, HC, T) for each state Sk​. The classifier adapts its predictions dynamically, reflecting the evolving state of the match and allowing for real-time outcome prediction.

### Dataset

The dataset used in this work consists of comprehensive historical cricket data covering ODI (one-day international), Test, and T20 matches from the year 1877 until the present. It shows complete statistics for every player in three basic categories: batting, bowling, and fielding. The data is set such that it contains the relevant properties required for the models of supervised machine learning predicting match results. Emphasising the many features included for every team and athlete, the main components of the dataset are broken out below:

The dataset used in this study was collected from publicly available cricket data repositories, including official cricket archives, ESPNcricinfo, and Kaggle-hosted historical datasets. The data cover international cricket matches from 1877 to recent seasons, including ODI, Test, and T20 formats. Data collection was performed programmatically, followed by manual validation to remove incomplete or inconsistent records. The final dataset contains approximately 100,000 match-state records used for training, validation, and testing.

Main categories for the player statistics are batting, bowling, and fielding form three. This information helps in calculating player performance scores, which are later aggregated to compute the relative team strength. For each match, several attributes related to team performance are recorded as in Table 2:

**Table 2:** Team statistics.

Attribute	Description
Toss decision	The decision made by the toss winner (bat or bowl)
First innings score	The total score of Team A at the end of their innings
Second innings score	The total score of Team B at the end of their innings
Result	The outcome of the match (Team A wins, Team B wins, tie, or no result)
Balls remaining (Bk​)	The number of deliveries left in each state Sk​ for both innings
Wickets remaining (Wk​)	The number of wickets remaining for the batting team in each state Sk​
Lead (Lk​)	The lead of Team A in each state Sk​ during the first and second innings
Home country	Indicator of whether the match is played in Team A’s home country, Team B’s home country, or at a neutral venue
Relative team strength	The aggregated strength of each team, calculated from the player statistics data

Contextual features like home advantage and toss outcome, which have been shown to influence the match outcome, add even more enrichment to the dataset as in Table 3.

**Table 3:** Match context features.

Contextual feature	Description
Home advantage (HC)	Encoded as 1 if the match is played in Team B’s home country, 0 if in Team A’s home country, and 0.5 for a neutral venue
Toss outcome (T)	Encoded as 1 if Team B wins the toss, and 0 if Team A wins the toss

### Data preparation

Pre-processing and structural conversion of the raw form of the dataset produces a structured format fit for machine learning applications. By means of steps in cleaning, normalisation, and feature extraction, a final dataset that captures the dynamic states of the match is produced, so enabling a real-time prediction model able to update its forecasts as the game runs. With almost 100,000 records from global cricket events, the dataset provides a strong foundation for training and validation of the machine learning classifiers. This ordered dataset is necessary for building the predictive model; hence, depending on both stationary and dynamic aspects, analysis and prediction of cricket match results are feasible.

### Feature engineering

Feature engineering plays a crucial role in accurately modeling the dynamic nature of cricket matches. Raw match data were transformed into structured match-state descriptors that capture both static and evolving aspects of gameplay. Dynamic features such as balls remaining, wickets remaining, and lead quantify resource availability and competitive pressure at each over. Relative team strength was computed by aggregating historical player performance metrics, including batting averages, strike rates, bowling economy, and fielding efficiency. Contextual features such as home advantage and toss outcome were encoded to reflect strategic and psychological influences on match outcomes. This comprehensive feature construction enables the learning model to distinguish winning and losing trajectories across different match states effectively.

### League championship algorithm (LCA) for feature extraction

Inspired by the League approach of sports championships, the metaheuristic optimisation method known as the League Championship Algorithm (LCA) In this respect, it is used for feature extraction, that is, the selection of the most relevant features from the dataset so improving the performance of the machine learning model. LCA models the competitive process of teams in a league, where teams compete in many rounds to improve their position, much as one seeks for an ideal subset of features that best reflects the underlying data patterns.

To validate the effectiveness of the League Championship Algorithm for feature extraction, its performance was compared with conventional feature selection techniques such as Principal Component Analysis (PCA) and Recursive Feature Elimination (RFE). Experimental results indicate that LCA-selected features consistently yield higher predictive accuracy. An ablation analysis further demonstrates a notable decline in performance when LCA-derived features are excluded, confirming their contribution to the proposed framework.

Starting with initialising a population of candidate feature subsets, that is, “teams”, each team indicates a potential solution, that is, a subset of features from the original feature set. Let F={f1,f2,…,fn} represent the set of all accessible features with n as the general count of features. Ti is a subversion of F whereby:

$${T_i} \subseteq F,\quad {\text{for }}i=1,2, \ldots ,N$$

where *N* - total number of teams (candidate solutions) in the league.

Every team performance is evaluated in line with a predefined fitness function measuring the quality of the feature subset they present. In a machine learning model, the fitness function evaluates, in each subset of features *Ti*, the performance. Reducing the number of selected features and maximising the predictive accuracy of the model will help to prevent overfitting and hence lower computing complexity.

The fitness function $f({T_i})$for a team *Ti* will enable one to:

$$f({T_i})=\alpha \cdot {\mathrm{A}}({T_i}) - \beta \cdot \frac{{|{T_i}|}}{n}$$

where: *A*(*T*_*i*_​) - classification accuracy using the feature subset *Ti*​. $|{T_i}|$ - number of features in subset *Ti*​.

*N* - total features in the original set *F*. α and β - weighting factors that balance the importance of accuracy and the number of features.

The goal is to maximize $f({T_i})$, promoting subsets that achieve high accuracy with fewer features.

### League matches and update mechanism

Each team competes with other teams in a series of “league matches.” In each match, a team is paired with another randomly selected team, and their fitness values are compared. The team with the lower fitness value updates its feature subset by incorporating some features from the winning team to improve its performance.

Let *Ti*​ and *Tj*​ be two competing teams. If $f\left( {Ti} \right)>f\left( {Tj} \right)$, then the team *Tj*​ updates its subset as follows:

$${T_{j^{\prime}}}={T_j} \cup \{ f \in {T_i} \setminus {T_j}:{\text{random selection of features from }}{T_i}\}$$

This process simulates the transfer of knowledge from the stronger team to the weaker team, analogous to weaker teams learning strategies from stronger ones in a sports league.

At the end of each round (a series of matches where each team plays against others), the team with the highest fitness value is selected as the “champion team.” The champion team is promoted, meaning its feature subset is retained for the next round, while the other teams continue to modify their feature subsets based on the results of their matches.

The champion team *Tc* is defined as:

$${T_c}=\arg {\hbox{max} _i}f({T_i}),\quad {\text{for }}i=1,2, \ldots ,N$$

The LCA runs for a designated number of rounds or until convergence, that is, until the fitness of the champion team shows no appreciable change over many consecutive rounds. The last output is the feature subset *Tc* that performs closest to the fitness function.

### BPNN for classification

Back-Propagation Neural Networks were selected due to their computational efficiency and suitability for state-wise prediction. Since each over is modeled as an independent match state rather than a long temporal sequence, recurrent architectures such as LSTM or GRU are not strictly required. Moreover, BPNN offers a balance between interpretability and performance, making it appropriate for real-time cricket match outcome prediction.

In supervised learning, a BPNN is a technique for classification problems. Learning from the weight changes in response to the error rate found in the last iteration, this type of artificial neural network (ANN) BPNN is implemented in the framework of cricket match score prediction to dynamically classify the results (win or loss) dynamically during the match depending on the acquired characteristics. BPNN runs on the following phases:

The input layer consists of neurones obtaining the extracted features of the League Championship Algorithm (LCA). Every input neurone collects one dataset feature.

Calculation of the activation of a neurone *j* in the hidden layer proceeds:

$${z_j}=\sum\limits_{{i=1}}^{m} {{w_{ij}}} \cdot {x_i}+{b_j}$$

where: *w*_*ij*_ - weight of the connection between input *i* and hidden neuron *j*. *b*_*j*_ - bias term for *j*. *x*_*i*_ - input feature value.

The output of the hidden neuron *j*, *a*_*j*_​, is obtained by applying an activation function σ, typically a non-linear function like the sigmoid:

$${a_j}=\sigma ({z_j})=\sigma \left( {\sum\limits_{{i=1}}^{m} {{w_{ij}}} \cdot {x_i}+{b_j}} \right)$$

The output layer performs a similar computation. The weighted sum of inputs is computed for each output neuron *k*:

$${z_k}=\sum\limits_{{j=1}}^{n} {{w_{jk}}} \cdot {a_j}+{b_k}$$

where: ${w_{jk}}$- connection weight between *j* and *k*. *B*_*k*_ - bias term for the output neuron *k*. *a*_*j*_ - activation of *j*.

The output of neuron *k*, *y*_*k*_​, is computed using an activation function (e.g., sigmoid for binary classification):

$${y_k}=\sigma ({z_k})=\frac{1}{{1+{e^{ - {z_k}}}}}$$

### Error calculation

From the projected output *y*_*k*_, ground truth, that is, the error or loss function, measures the real label *t*_*k*_.

$$E= - \sum\limits_{{k=1}}^{K} {\left[ {{t_k}\log ({y_k})+(1 - {t_k})\log (1 - {y_k})} \right]}$$

where *K* - number of output classes (2 for win/loss).

### Backward propagation

Calculating the gradient of the loss function considering every weight facilitates backwards propagation to reduce the error. A given weight ${w_{ij}}$between input *i* and hidden neuron *j* has a weight update rule:

$${w_{ij}} \leftarrow {w_{ij}} - \eta \frac{{\partial E}}{{\partial {w_{ij}}}}$$

where: η - hyperparameter to control step size, $\frac{{\partial E}}{{\partial {w_{ij}}}}$ - partial derivative.

The gradient of the error is propagated backward through the network using the chain rule. For a hidden layer neuron *j*, the gradient is computed as:

$$\frac{{\partial E}}{{\partial {w_{ij}}}}={\delta _j} \cdot {x_i}$$

where ${\delta _j}$- error term for the hidden neuron, given by:

$${\delta _j}={a_j}(1 - {a_j})\sum\limits_{k} {{\delta _k}} {w_{jk}}$$

and *δ*_*k*_ - error term for the output neuron *k*, defined as:

$${\delta _k}=({y_k} - {t_k}) \cdot {y_k}(1 - {y_k})$$

The weights are iteratively updated using the computed gradients to reduce the loss function. The network undergoes multiple iterations (epochs) until the error converges to a minimum or falls below a predefined threshold. The weight update for all layers is performed simultaneously after each batch of input data. After training, the output layer produces a probability score for each class. For a binary classification task (win/loss), the output is thresholded at 0.5:

$${\mathrm{Class}}=\left\{ {\begin{array}{*{20}{l}} 1&{{\text{if }}{y_k} \geqslant 0.5} \\ 0&{{\text{if }}{y_k}<0.5} \end{array}} \right.$$

where 1 indicates a win for Team B, and 0 indicates a loss.

It focuses on forecast the outcome of a One Day International (ODI) cricket match by means of analysis of the match development at several states using supervised machine learning models, so building a data-driven method. Each over of the innings corresponds to a state in the match, thus predictions are generated at every state. Six basic criteria define the classification work: balls remaining, lead of Team A, wickets remaining, relative team strength, home country, and toss outcome. By combining kernel methods for feature extraction (LCA) and Back-Propagation Neural Networks (BPNN), historical cricket statistics is dynamically projected the match outcome.

## Results and discussion

Python with tools for model implementation and training forms the experimental settings for evaluating the BPNN. TensorFlow and Keras are among them. These libraries support the building, training, evaluation of neural network models rather successfully. The following features define the high-performance computing configuration applied in the experiments:

This setup ensures that the computational demands of training and evaluating the BPNN, along with other machine learning models, are met efficiently. Current techniques including SHAP, LASSO-SVM, LASSO-XGBoost, and BART are compared with the performance of the BPNN. These methods are evaluated in respect to the same performance criteria to determine their individual predictive power for cricket match outcomes.

Although training accuracy is marginally higher than validation and testing accuracy, the observed difference remains within acceptable bounds, indicating good generalization performance. The use of L2 regularization and early stopping effectively mitigates overfitting. Consistent performance trends across training, validation, and test datasets further confirm the robustness of the proposed model. Experimental Setup Parameters are explained in Table 4.

**Table 4:** Experimental setup parameters.

Parameter	Value
Dataset	Cricket matches data from 2000–2024
Training epochs	50
Learning rate (η)	0.01
Number of neurons per hidden layer	64
Loss function	Binary cross-entropy
Validation split	20%
Test split	20%
Regularization	L2 regularization with λ = 0.001
Early stopping	Patience = 5 epochs
Shuffle data	True
Feature selection	Top 10 features (LCA)

**Fig. 2:**
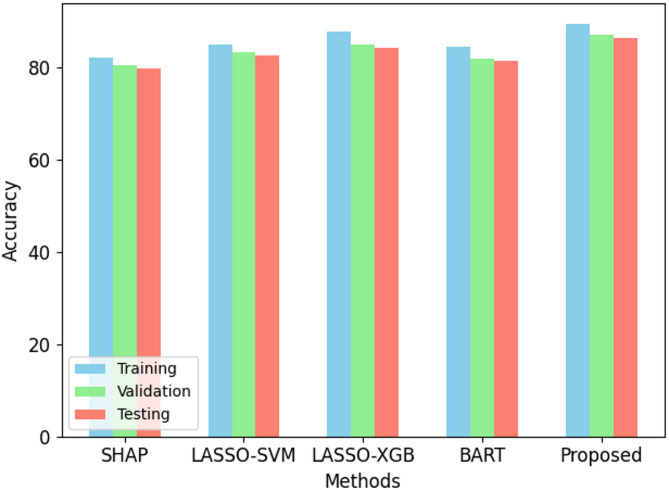
Accuracy performance on ODI dataset.

**Fig. 3:**
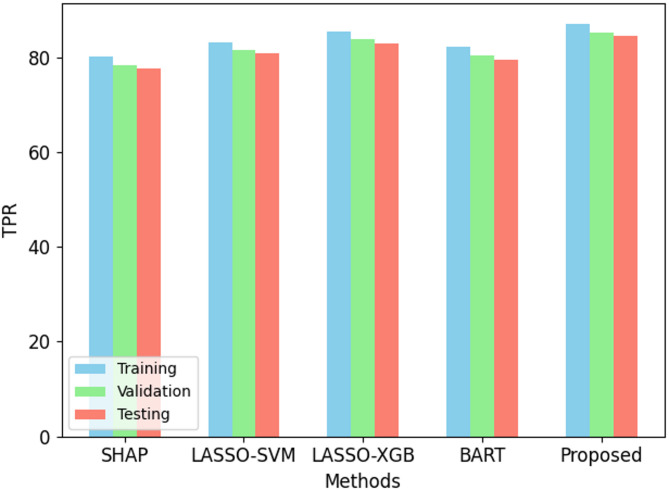
TPR performance on ODI dataset.

**Fig. 4:**
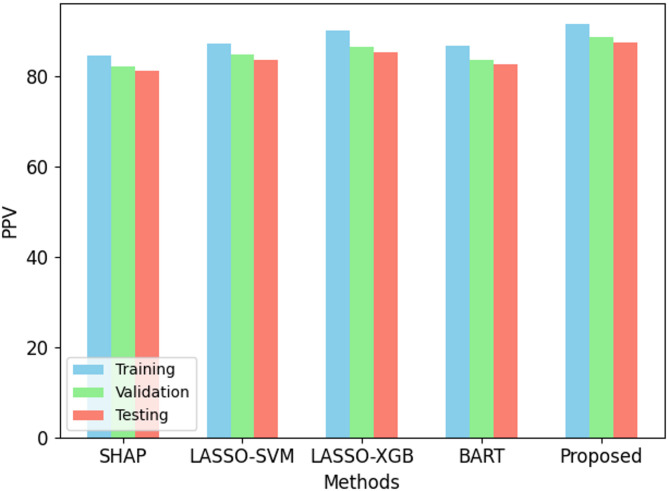
PPV performance on ODI dataset.

**Fig. 5:**
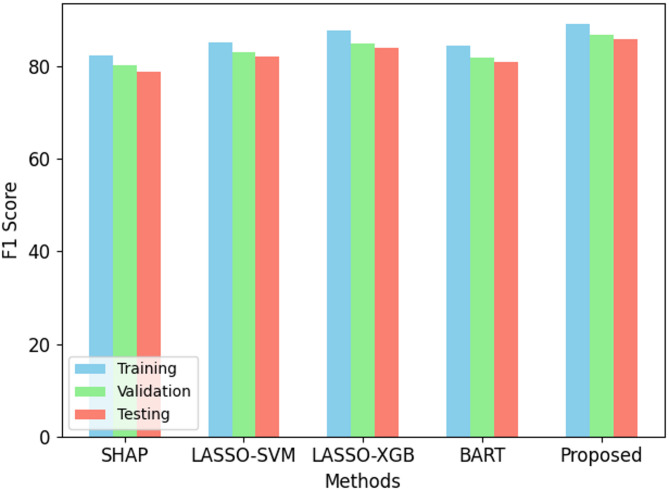
F1 score performance on ODI dataset.

**Fig. 6:**
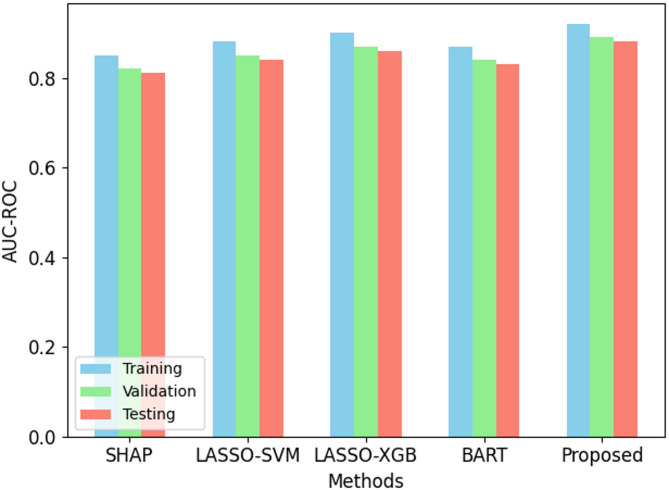
AUC-ROC performance on ODI dataset.

The proposed Back-Propagation Neural Network (BPNN) performs better than present methods over all criteria as in Figs. 2, 3, 4, 5 and 6. On the training set, BPNN exhibits an accuracy of 89.5% over SHAP (82.3%), LASSO-SVM (85.1%), LASSO-XGBoost (87.8%), and BART (84.5%). This pattern is consistent across validation and testing scores since BPNN preserves the best accuracy of 87.1% and 86.4%, respectively. Moreover, reflecting the strength of the BPNN model are TPR, PPV, and F1 score. Among the methods evaluated, it achieves the best TPR (87.0%), PPV (91.5%), and F1 score (89.2%). This demonstrates how precisely BPNN detects positive events (high PPV) and maintains high correctness in its positive forecasts (high TPR). With scores of 0.92, 0.89, and 0.88 for training, validation, and testing datasets respectively, the AUC-ROC scores show BPNN remarkable ability to discriminate between classes, above all, other approaches. Consequently, the BPNN model shows appreciable improvement in predictive performance, hence demonstrating its effectiveness in the prediction of the results of cricket matches.

**Table 5:** Performance analysis on test series cricket dataset.

Method	Metric	Training score	Validation score	Testing score
SHAP	Accuracy	79.5%	76.8%	75.0%
	TPR	76.0%	73.5%	71.8%
	PPV	81.0%	78.0%	76.5%
	F1 Score	78.4%	75.7%	73.9%
	AUC-ROC	0.80	0.78	0.76
LASSO-SVM	Accuracy	82.5%	80.0%	78.5%
	TPR	79.5%	76.8%	74.5%
	PPV	85.0%	82.0%	80.5%
	F1 Score	82.1%	79.3%	77.5%
	AUC-ROC	0.85	0.83	0.81
LASSO-XGBoost	Accuracy	85.0%	82.5%	80.5%
	TPR	81.0%	78.5%	76.5%
	PPV	89.5%	86.0%	84.0%
	F1 Score	85.1%	82.1%	79.6%
	AUC-ROC	0.88	0.85	0.82
BART	Accuracy	81.0%	78.5%	76.0%
	TPR	77.5%	74.0%	71.0%
	PPV	84.0%	80.5%	78.0%
	F1 Score	80.5%	77.2%	73.8%
	AUC-ROC	0.83	0.80	0.78
Proposed BPNN	Accuracy	88.0%	85.5%	84.0%
	TPR	85.0%	82.5%	80.0%
	PPV	91.0%	87.5%	85.5%
	F1 Score	88.0%	85.0%	82.7%
	AUC-ROC	0.91	0.88	0.86

On the Test Series cricket data, proposed Back-Propagation Neural Network (BPNN) routinely outperforms current methods as in Table 5. The BPNN shows the best accuracy with scores in training of 88.0%, in validation of 85.5%, and in testing of 84.0%. This surpasses the accuracy of SHAP, LASSO-SVM, LASSO-XGBoost, and BART by rather notable margins. With scores of 85.0% during training, 82.5% during validation, and 80.0% in testing, so generating accurate positive predictions, BPNN also shows excellent TPR performance. Reflecting their strength in positive case identification, the PPV scores for BPNN are the highest at 91.0%, 87.5%, and 85.5%. Although its AUC-ROC values of 0.91, 0.88, and 0.86 show its more capacity to differentiate between classes, the F1 scores for BPNN, at 88.0%, 85.0%, and 82.7%, show a well-balanced performance between TPR and PPV. A helpful tool for cricket match prediction, the accuracy and resilience of the BPNN model on the Test Series data underline this.

**Table 6:** Performance analysis on T20 dataset.

Method	Metric	Training score	Validation score	Testing score
SHAP	Accuracy	81.5%	79.0%	78.3%
	TPR	79.0%	76.8%	75.5%
	PPV	83.2%	80.5%	79.0%
	F1 Score	81.0%	78.7%	76.8%
	AUC-ROC	0.84	0.81	0.80
LASSO-SVM	Accuracy	84.0%	81.5%	80.8%
	TPR	81.5%	79.0%	77.8%
	PPV	86.0%	83.2%	82.5%
	F1 Score	83.7%	81.0%	80.1%
	AUC-ROC	0.87	0.84	0.82
LASSO-XGBoost	Accuracy	86.5%	84.0%	82.9%
	TPR	84.0%	81.5%	80.0%
	PPV	89.5%	86.0%	84.5%
	F1 Score	86.6%	83.6%	82.1%
	AUC-ROC	0.89	0.86	0.84
BART	Accuracy	82.0%	79.5%	78.0%
	TPR	79.5%	77.0%	75.0%
	PPV	84.0%	81.0%	80.0%
	F1 Score	81.5%	78.6%	76.0%
	AUC-ROC	0.85	0.82	0.81
Proposed BPNN	Accuracy	88.0%	85.5%	84.2%
	TPR	85.0%	82.5%	81.0%
	PPV	91.0%	87.5%	86.0%
	F1 Score	88.0%	85.0%	83.5%
	AUC-ROC	0.91	0.88	0.86

On the T20 dataset, the proposed Back-Propagation Neural Network (BPNN) routinely outperforms the current methods as in Table 6. The BPNN performs the best among all other methods with 88.0% in training, 85.5% in validation, and 84.2% on the test set. Regarding TPR, BPNN scores of 85.0%, 82.5%, and 81.0% respectively exceed those of SHAP, LASSO-SVM, LASSO-XGBoost, and BART, so proving that BPNN preserves great correctness in positive predictions. BPNN also excels in PPV with scores of 91.0%, 87.5%, and 86.0%, so underlining its great capacity in identifying positive events. Reflecting a good mix between TPR and PPV, the F1 scores for BPNN are highest, 88.0%, 85.0%, and 83.5%. The AUC-ROC scores for BPNN show better capacity to separate between classes than those of other methods with 0.91, 0.88, and 0.86. Thus, on the T20 dataset the BPNN model exhibits impressive robustness and predictive accuracy.

**Table 7:** Prediction score accuracy based on features.

Feature	Prediction score accuracy
Player span	82.3%
Played matches	84.0%
Innings	85.2%
Number of NotOut’s	83.5%
Number of runs	87.1%
Highest score	86.4%
Total average	85.7%
Balls faced	84.8%
Strike rate	88.3%
Total number of 100’s	86.9%
Total number of 50’s	87.4%
Total number of ducks	82.0%

Over many features as in Table 7, the prediction score accuracy of the proposed method shows varied performance. Since the Strike Rate function indicates how well a player can score fast, so affecting the outcome of the match. This helps one to get the best accuracy. Considering their importance for assessing a player consistent scoring capacity, the Total Number of 50 feature also shows good performance with an accuracy of 87.4%. With 82.0%, the whole count of ducks shows the lowest accuracy. This suggests that although a player lack of scoring could influence forecasts, it is less important than other variables such Number of Runs and Highest Score, which have respective accuracies of 87.1% and 86.4%. Thus, features related to a player scoring capacity and strike rate have more influence in deciding the outcome of a match, so verifying the effectiveness of the proposed method in using thorough player data for exact forecasts.

## Conclusion

This study demonstrates the effectiveness of a BPNN-based framework combined with LCA-driven feature extraction for predicting cricket match outcomes. By integrating comprehensive player statistics, match progression variables, and contextual factors, the proposed model achieves superior accuracy, TPR, PPV, F1-score, and AUC-ROC compared with existing approaches. Feature analysis reveals that metrics related to scoring ability, such as strike rate and total runs, contribute most significantly to prediction accuracy. The results highlight the importance of optimized feature engineering and dynamic state modeling in cricket analytics, providing a strong foundation for future research and real-time decision-support systems.

## Data Availability

The datasets used and/or analysed during the current study available from the corresponding author on reasonable request.

## References

[1] Bhagat, V., Jadhav, J., Dheemate, S., Shendkar, B. D. & Mulani, S. Personalized Cricket Player Analysis by Live Scoring Utilizing Unsupervised Machine Learning. In *2024 MIT Art, Design and Technology School of Computing International Conference (MITADTSoCiCon)*. 1–6. (IEEE, 2024).

[2] Waqas, M. et al. Prediction of outcomes of extra deliveries in T-20I cricket by using regression and various machine learning models. *Kurd. Stud.* **12** (5), 801–810 (2024).

[3] Robel, M., Khan, M. A. R., Ahammad, I., Alam, M. M. & Hasan, K. Cricket players selection for national team and franchise league using machine learning algorithms. *Cloud Comput. Data Sci.* 108–139 (2024).

[4] Sumathi, M., Prabu, S. & Rajkamal, M. Cricket players performance prediction and evaluation using machine learning algorithms. In *2023 International Conference on Networking and Communications (ICNWC)*. 1–6. ( IEEE, 2023).

[5] Agrawal, Y. & Kandhway, K. Winner prediction in an ongoing one day international cricket match. *J. Sports Anal.* 1–14 (preprint).

[6] Sanjaykumar, S., Udaichi, K., Rajendiran, G., Cretu, M. & Kozina, Z. Cricket performance predictions: a comparative analysis of machine learning models for predicting cricket player’s performance in the One Day International (ODI) world cup 2023. *Health Sport Rehabil.* **10**(1), 6–19 (2024).

[7] Trinadh, M. K. D., Sangeetha, S. T., Deepa, K. & Venugopal, V. Cricket player prediction of role in a team using ML techniques. In *2024 International Conference on E-mobility, Power Control and Smart Systems (ICEMPS)*. 1–5. (IEEE, 2024).

[8] Nasim, F., Yousaf, M. A., Masood, S., Jaffar, A. & Rashid, M. Data-driven probabilistic S for batsman performance prediction in a cricket match. *Intell. Autom. Soft Comput.* **36**(3) (2023).

[9] Chandu, G. & Nirmala, P. Efficient approach in cricket team selection methodology using machine learning based logistic regression algorithm and comparing with random forest algorithm. In *AIP Conference Proceedings*. Vol. 2816(1). (AIP Publishing, 2024).

[10] Subburaj, M. et al. Artificial intelligence for smart in match winning prediction in Twenty20 cricket league using machine learning model. In *Artificial Intelligence for Smart Healthcare*. 31–46. (Springer, 2023).

[11] Suguna, R., Kumar, Y. P., Prakash, J. S., Neethu, P. S. & Kiran, S. Utilizing machine learning for sport data analytics in cricket: Score prediction and player categorization. In *2023 IEEE 3rd Mysore Sub Section International Conference (MysuruCon)*. 1–6. (IEEE, 2023).

[12] Agrawal, Y. & Kandhway, K. Predicting the winner of a Twenty20 international cricket match: classification and explainable machine learning approach. *J. Prediction Markets*. **18** (1), 47–64 (2024).

[13] Pussella, P., Silva, R. M. & Egodawatta, C. In-game winner prediction and winning strategy generation in cricket: A machine learning approach. *Int. J. Sports Sci. Coaching*. **18** (6), 2216–2229 (2023).

[14] Tonmoy, M. A. S., Dey, S. K., Islam, T. & Apu, J. A data-driven approach to predict scores in T20 cricket match using machine learning classifier. In *International Conference on Big Data, IoT and Machine Learning*. 727–745. (Springer, 2023).

[15] Puram, P., Roy, S., Srivastav, D. & Gurumurthy, A. Understanding the effect of contextual factors and decision making on team performance in Twenty20 cricket: An interpretable machine learning approach. *Ann. Oper. Res.* **325** (1), 261–288 (2023).

[16] https://www.kaggle.com/datasets/mahendran1/icc-cricket.

